# Acute Chest Pain Related to Pericardial Fat Necrosis

**DOI:** 10.1155/2016/1948325

**Published:** 2016-05-03

**Authors:** Gilbert R. Ferretti, Dominique Rigaud

**Affiliations:** Department of Radiology, Grenoble Alpes University, CHU Grenoble, BP 217, 38043 Grenoble Cedex 09, France

An otherwise healthy 56-year-old woman (BMI, 31 kg/m^2^) presented to our emergency department with acute nonradiating left-sided chest pain. She had no associated dyspnea, fever, or other clinical findings. An electrocardiogram and serum troponin level were unremarkable. Nonenhanced computed tomography (CT) of the chest (Figure 1) showed a left anterior low-attenuation (−80 HU) pericardial mass with high attenuation strands measuring 47 mm in diameter, surrounded by an irregular capsule (arrow), and with an associated small pleural effusion (arrowhead). Coronal reformation CT confirmed no connection with infradiaphragmatic tissues (Figure 2). Contrast-enhanced CT (Figure 3) ruled out pulmonary embolism and aortic dissection but confirmed the typical appearance of pericardial fat necrosis. With nonsteroidal anti-inflammatory treatment the pain disappeared. On follow-up CT after 3 months (Figure 4), the mass was still present but significantly reduced in size (28 mm) (arrow).

Pericardial (or epipericardial or paracardial) fat necrosis is a rare benign cause of sudden chest pain that can mimic more severe diseases such as myocardial infarction, pulmonary embolism, or pericarditis [1]. Patients with pericardial fat necrosis have no associated systemic symptoms, however, and laboratory tests and echocardiography are unremarkable [1]. Chest radiography may show increased attenuation in a juxtacardiac location and pleural effusion. Computed tomography, the key diagnostic test, shows a mass with fat attenuation located within the anterior mediastinal fat adjacent to the pericardium and surrounded by a ring of increased density that is enhanced after contrast media injection. There is often an associated ipsilateral pleural effusion [2].

It is important to recognize these characteristic CT findings of pericardial fat necrosis to avoid unnecessary exploratory thoracotomy for a benign lesion [3]. James et al. [4] reported a case of mild FDG uptake at PET/CT in a case of pericardial fat necrosis, representing the inflammatory process.

The differential diagnosis for a pericardial mass with fat attenuation includes primary fatty tumors (lipoma, liposarcoma, teratoma, and thymolipomas), diaphragmatic hernias with abdominal fat occupying the cardiophrenic space, and mediastinitis.

## Figures and Tables

**Figure 1:**
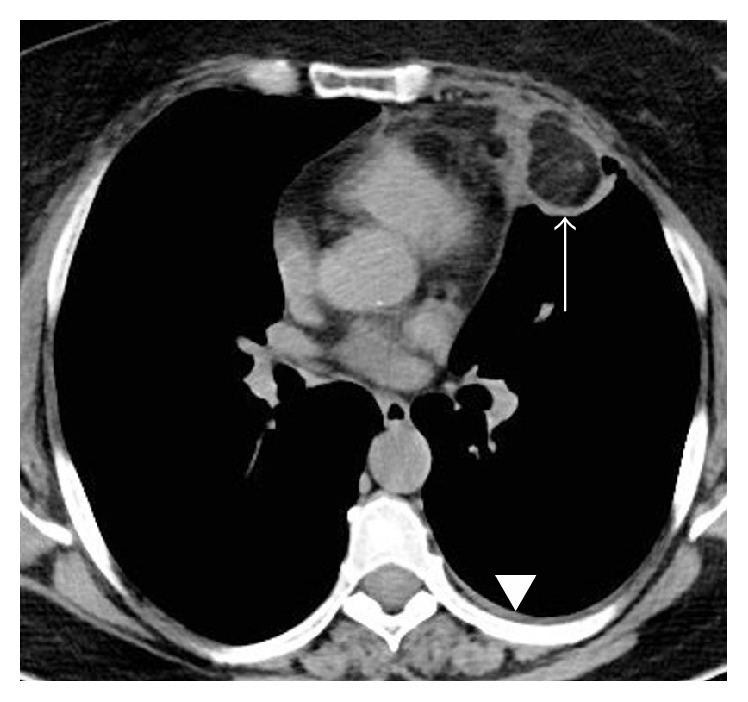
CT scan without contrast enhancement showing a left anterior low-attenuation (−80 HU) pericardial mass with high attenuation strands surrounded by an irregular capsule (arrow) and a small pleural effusion (arrowhead).

**Figure 2:**
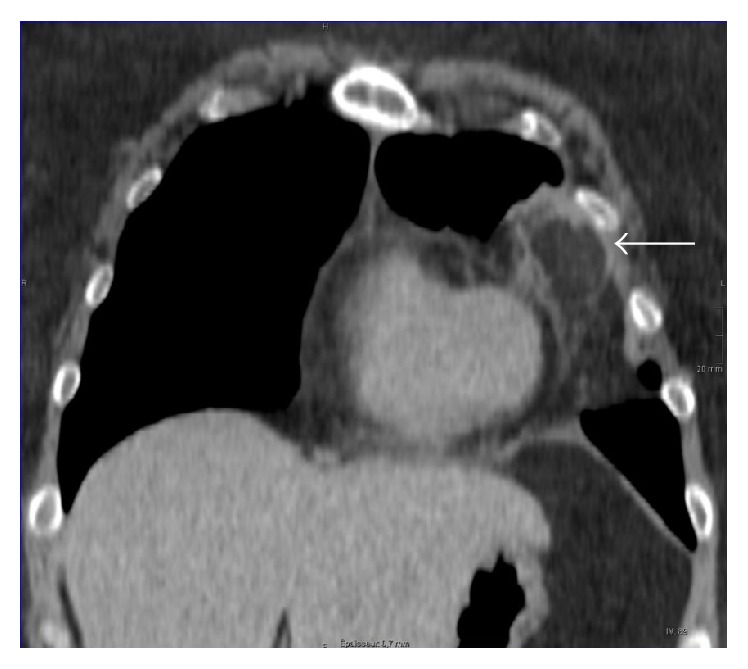
Coronal reformatted CT showing no connection between the pericardial mass and infradiaphragmatic fatty tissues.

**Figure 3:**
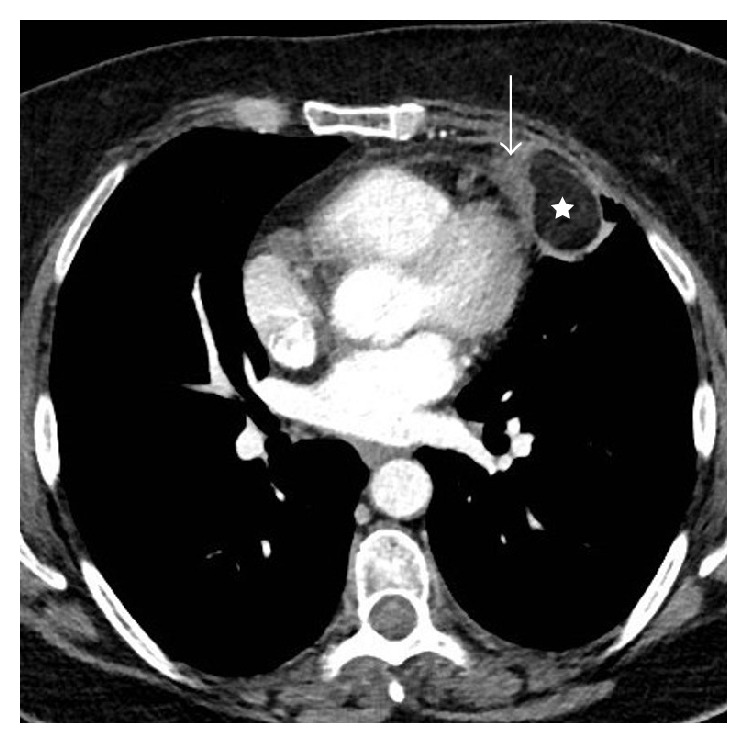
Contrast-enhanced CT showing that the central fatty attenuation of the mass is not enhanced (star); the capsule shows a 50 HU increase in density (arrow).

**Figure 4:**
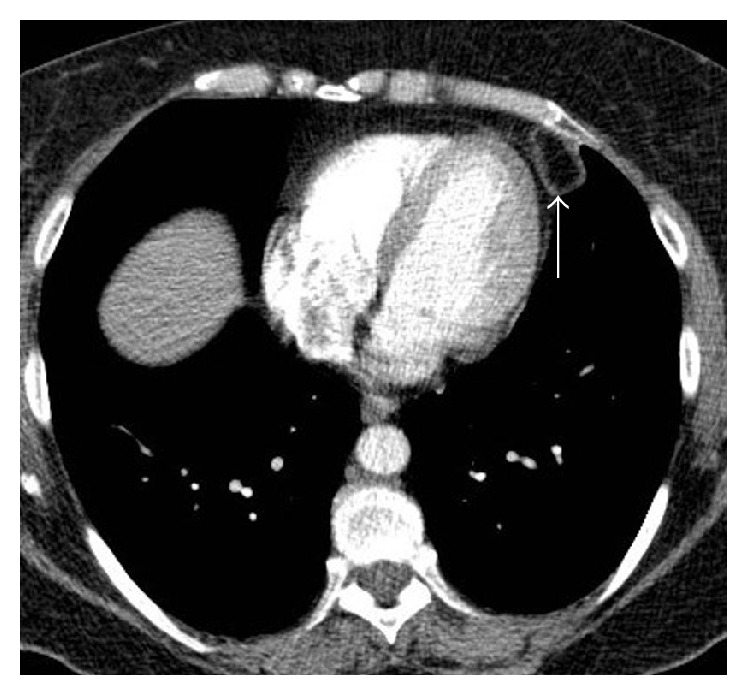
CT scan after 3 months showing a significant reduction in the size of the mass (arrow).

## Additional Points

1. Pericardial fat necrosis is a rare cause of acute anterior chest pain.
2. CT shows typical fatty mass surrounded by a ring of contrast-enhanced attenuation.
3. Surgery should be avoided when the typical CT appearances are present.
